# Supplementary material for: Inter-sleep stage variations in corrected QT interval differ between obstructive sleep apnea patients with and without stroke history
Source: PLoS One. 2022 Dec 1;17(12):e0278520. doi: 10.1371/journal.pone.0278520 (PMC9714836; doi:10.1371/journal.pone.0278520)
Supplement: S1 Checklist — (PDF) [file pone.0278520.s001.pdf]

STROBE Statement—checklist of items that should be included in reports of observational studies

	Item No	Recommendation
**Title and abstract**	1	(*a*) Indicate the study's design with a commonly used term in the title or the abstract<br>***Retrospective design in the abstract (page 2)***
		(*b*) Provide in the abstract an informative and balanced summary of what was done and what was found<br>***Presented on page 2***
**Introduction**		
Background/rationale	2	Explain the scientific background and rationale for the investigation being reported<br>***Explained on pages 3 and 4***
Objectives	3	State specific objectives, including any prespecified hypotheses<br>***Stated on page 4***
**Methods**		
Study design	4	Present key elements of study design early in the paper<br>***Presented in the abstract, at the end of the introduction section (page 4), and in the methods section (pages 4-6)***
Setting	5	Describe the setting, locations, and relevant dates, including periods of recruitment, exposure, follow-up, and data collection<br>***Presented on pages 4 and 5***
Participants	6	(*a*) *Cohort study*—Give the eligibility criteria, and the sources and methods of selection of participants. Describe methods of follow-up<br>***Not applicable***<br>*Case-control study*—Give the eligibility criteria, and the sources and methods of case ascertainment and control selection. Give the rationale for the choice of cases and controls<br>***Presented in the methods section, page 4***<br>*Cross-sectional study*—Give the eligibility criteria, and the sources and methods of selection of participants<br>***Not applicable***
		(*b*) *Cohort study*—For matched studies, give matching criteria and number of exposed and unexposed<br>***Not applicable***<br>*Case-control study*—For matched studies, give matching criteria and the number of controls per case<br>***Presented on page 4***
Variables	7	Clearly define all outcomes, exposures, predictors, potential confounders, and effect modifiers. Give diagnostic criteria, if applicable<br>***Defined on pages 5-6***
Data sources/ measurement	8*	For each variable of interest, give sources of data and details of methods of assessment (measurement). Describe comparability of assessment methods if there is more than one group<br>***Defined on pages 5-6***
Bias	9	Describe any efforts to address potential sources of bias<br>***Described in the discussion section (pages 9-13)***
Study size	10	Explain how the study size was arrived at

		Explained in the methods section (pages 4-5)
Quantitative variables	11	Explain how quantitative variables were handled in the analyses. If applicable, describe which groupings were chosen and why<br>***Described on pages 4-6***
Statistical methods	12	(*a*) Describe all statistical methods, including those used to control for confounding<br>***Described on pages 5-6***
		(*b*) Describe any methods used to examine subgroups and interactions<br>***Described on page 5-6***
		(*c*) Explain how missing data were addressed<br>***Not applicable***
		(*d*) *Cohort study*—If applicable, explain how loss to follow-up was addressed<br>***Not applicable***<br>*Case-control study*—If applicable, explain how matching of cases and controls was addressed<br>***Described in the methods section, pages 4-6***<br>*Cross-sectional study*—If applicable, describe analytical methods taking account of sampling strategy<br>***Not applicable***
		(*e*) Describe any sensitivity analyses<br>***Not applicable***

Results

Participants	13*	(a) Report numbers of individuals at each stage of study—eg numbers potentially eligible, examined for eligibility, confirmed eligible, included in the study, completing follow-up, and analysed<br>***Described in the methods (page 4) and results (page 7) sections***
		(b) Give reasons for non-participation at each stage<br>***Described on pages 4-6***
		(c) Consider use of a flow diagram<br>***Not applicable***
Descriptive data	14*	(a) Give characteristics of study participants (eg demographic, clinical, social) and information on exposures and potential confounders<br>***Given in Table 1 (page 7)***
		(b) Indicate number of participants with missing data for each variable of interest<br>***Not applicable***
		(c) *Cohort study*—Summarise follow-up time (eg, average and total amount)<br>***Not applicable***
Outcome data	15*	*Cohort study*—Report numbers of outcome events or summary measures over time<br>***Not applicable***
		Case-control study—Report numbers in each exposure category, or summary measures of exposure<br>***Given in Table 2 (page 7)***
		Cross-sectional study—Report numbers of outcome events or summary measures<br>***Not applicable***
Main results	16	(*a*) Give unadjusted estimates and, if applicable, confounder-adjusted estimates and their precision (eg, 95% confidence interval). Make clear which confounders were adjusted for and why they were included

		No adjusting required
		(*b*) Report category boundaries when continuous variables were categorized<br>***Not applicable***
		(*c*) If relevant, consider translating estimates of relative risk into absolute risk for a meaningful time period<br>***Not applicable***
Other analyses	17	Report other analyses done—eg analyses of subgroups and interactions, and sensitivity analyses<br>***Not applicable***
**Discussion**		
Key results	18	Summarise key results with reference to study objectives<br>***Summarized on page 9***
Limitations	19	Discuss limitations of the study, taking into account sources of potential bias or imprecision. Discuss both direction and magnitude of any potential bias<br>***Discussed on pages 12-13***
Interpretation	20	Give a cautious overall interpretation of results considering objectives, limitations, multiplicity of analyses, results from similar studies, and other relevant evidence<br>***Interpretation conducted throughout discussion (pages 9-13)***
Generalisability	21	Discuss the generalisability (external validity) of the study results<br>***Discussed on pages 9-13***
**Other information**		
Funding	22	Give the source of funding and the role of the funders for the present study and, if applicable, for the original study on which the present article is based<br>***Reported through a separate statement at the time of submission***

*Give information separately for cases and controls in case-control studies and, if applicable, for exposed and unexposed groups in cohort and cross-sectional studies.

Note: An Explanation and Elaboration article discusses each checklist item and gives methodological background and published examples of transparent reporting. The STROBE checklist is best used in conjunction with this article (freely available on the Web sites of PLoS Medicine at http://www.plosmedicine.org/, Annals of Internal Medicine at http://www.annals.org/, and Epidemiology at http://www.epidem.com/). Information on the STROBE Initiative is available at www.strobe-statement.org.
