# Supplementary material for: Inter-sleep stage variations in corrected QT interval differ between obstructive sleep apnea patients with and without stroke history
Source: PLoS One. 2022 Dec 1;17(12):e0278520. doi: 10.1371/journal.pone.0278520 (PMC9714836; doi:10.1371/journal.pone.0278520)
Supplement: Attachment — Submitted filename: Response to Reviewers.pdf [file pone.0278520.s002.pdf]

Inter-sleep stage variations in corrected QT interval differ between obstructive sleep apnea patients with and without stroke history

Serajeddin Ebrahimian, Saara Sillanmäki, Salla Hietakoste, Brett Duce, Antti Kulkas, Juha Töyräs, Timo Leppänen, Jukka A. Lipponen, Samu Kainulainen

We want to thank the Academic Editor and the Reviewer for considering this original article to be published in the PLOS ONE journal. We also would like to thank the Reviewer for the constructive and helpful comments and suggestions, according to which we have revised the manuscript.

We have now listed all the comments, followed by clear and concise responses and when necessary, have included the corresponding action implemented in the revised manuscript with reference to modified text with page and line numbers. The page and line numbers are based on the revised manuscript with track changes. The modified text is highlighted in red in this response and the revised manuscript. The actions regarding the responses to the Reviewer can be found in the following.

We believe that the revised manuscript has improved significantly from the initial version based on the recommendations of the Reviewer and hope that you find it suitable for publication in the PLOS ONE journal.

Sincerely,
Serajeddin Ebrahimian, M.Sc.
Corresponding Author
October 28th, 2022, Kuopio, Finland
Sleep Technology and Analytics Research Group
The Department of Applied Physics // University of Eastern Finland
Diagnostic Imaging Center // Kuopio University Hospital

RESPONSE TO THE REVIEWER #1

We want to sincerely thank the Reviewer for the constructive review of our manuscript. Below, we provide point-by-point responses to each comment made by the Reviewer and reference to the modified text. We believe that the manuscript has significantly improved based on the valuable comments given by the Reviewer.

Comment 1: Methods. It should be added how "randomly selected" is defined.

Author Response: Thank you for this important notification. For the control group, we listed all the patients with the inclusion criteria (apnea-hypopnea index (AHI) ≥ 15, no ventricular pacing, and total sleep time ≥ 4 hours in PSG) and used a random number generator ("randi" function in MATLAB R2021b [MathWorks Inc, MA, USA]) to select 18 patients.

To address this comment, the following changes have been made to the revised manuscript:

Methods: "The control group comprised ~~randomly selected~~ 18 (12 men) subjects with similar inclusion criteria but without a previous stroke history. The subjects in the control group were randomly selected using a random number generator ("randi" function in MATLAB R2021b [MathWorks Inc, MA, USA])."
Page 4, lines 84-87.

Comment 2: Methods. Please report the strobe statement.

Author Response: We want to thank the Reviewer for this comment. We have prepared the strobe statement as requested. It is provided as a separate file titled "Ebrahimian_etal_STROBE_checklist" as an attachment to the response to the reviewers' file.

Comment 3: Methods. It is not clear how patients were selected. I think that there is a risk of bias toward those patients (probably at high risk) who had all these data available.

Author Response: Thank you for this comment. The initial dataset of over 900 patients includes full in-lab PSGs and demographic information (e.g., age, BMI, comorbidities) from all the patients. Out of the initial dataset, 46 had a stroke history. Within these patients, 18 fulfilled the inclusion criteria (apnea-hypopnea index ≥ 15, no ventricular pacing, and total sleep time ≥ 4 hours in PSG). For the control group, subjects were selected from patients with similar inclusion criteria without stroke history. Subjects in the control group were randomly selected using a random number generator and without any human control to eliminate possible bias in selection. Thus, we believe there is no selection bias in our study. Our randomized patient selection for the control group was clarified in our response to Comment 1.

Comment 4: Methods. Tables should not be reported in the methods but in the results.

Author Response: We want to thank the Reviewer for this recommendation. We agree with the Reviewer that another possible location for Tables 1 and 2 is at the beginning of the Results section. Thus, Tables 1 and 2 have now been moved to the beginning of the results section in the revised manuscript.

To address this comment, the following changes have been made to the revised manuscript:

Methods: "~~The demographic data of the studied population are presented in Table 1.~~"
Page 4, lines 87-88.

Methods: "RR, QT, and QTc intervals were calculated for each ECG segment ~~were compared between sleep stages and the number of analyzed segments in each sleep stage is presented in Table 2~~."
Page 6, lines 117-118.

*Results: "*The demographic data of the studied population are presented in Table 1 and the number of analyzed segments in each sleep stage is presented in Table 2."
Page 7, lines 141-142.

Comment 5: Methods. It should be included why HR less than 30 should be defined as low.

Author Response: We want to thank the Reviewer for this important notification. A slow heart rate with rates around 40–50 beat per minute (BPM) is common and is considered normal during sleep [1]. Given that apnea occurrences are associated with lowered HR during apnea [2] it is expected that occasionally heart rate falls below 40 BPM during sleep in patients with sleep apnea. However, as the low technical quality of ECG recording can lead to erroneously low average HR values in segments due to missed R-peak detections, we considered an average HR of 30 BPM as a threshold to exclude low-quality segments. In our dataset, the segments had an average HR of 66 BPM with a standard deviation of 14.5 BPM. By applying this exclusion rule, 401 segments were excluded out of over 25000 segments due to low technical quality. Thus, we think that using the 30 BPM as a threshold to exclude possibly erroneous segments is feasible and does not jeopardize the conclusions made in this study.

To address this comment, the following changes have been made to the revised manuscript:

Methods: "ECG segments with ≥ 5% beat correction ~~and heart rate < 30~~ were excluded from the analysis to avoid the inclusion of low-quality segments. A slow heart rate (i.e. around 40-50 beats per minute [BPM]) during sleep is common [25] and can be further lowered due to possible transient decrease during apnea/hypopnea events [26]. However, as the low technical quality of ECG recording can lead to erroneously low HR values in segments due to missed R-peaks detection, we also considered an average HR of 30 BPM as a threshold for exclusion of low-quality segments."
Pages 6, lines 109-115.

References: The following two references were added, and the references list and reference numberings were updated accordingly.

25. Sessa F, Anna V, Messina G, Cibelli G, Monda V, Marsala G, et al. Heart rate variability as predictive factor for sudden cardiac death. Aging (Albany NY). 2018;10: 166–177. doi:10.18632/aging.101386

26. Guilleminault C, Connolly S, Winkle R, Melvin K, Tilkian A. Cyclical variation of the heart rate in sleep apnoea syndrome: Mechanisms, and usefulness of 24 h electrocardiography as a screening technique. Lancet (London, England). 1984;1: 126–131. doi:10.1016/s0140-6736(84)90062-x

---

AUTHORS MODIFICATION

Authors identified that a small correction need to be applied to the captions of the figures 1 and 2. The following changes have been made to the revised manuscript

Results: “n.s” changed to “n.s.”. **Page 8, line 159 and Page 9, line 175.**
